# Combination of colonoscopy and magnetic resonance enterography is more useful for clinical decision making than colonoscopy alone in patients with complicated Crohn's disease

**DOI:** 10.1371/journal.pone.0212404

**Published:** 2019-02-20

**Authors:** Shintaro Sagami, Taku Kobayashi, Nao Kikkawa, Satoko Umeda, Masaru Nakano, Takahiko Toyonaga, Shinji Okabayashi, Ryo Ozaki, Toshifumi Hibi

**Affiliations:** 1 Center for Advanced IBD Research and Treatment, Kitasato University Kitasato Institute Hospital, Minato-ku, Tokyo, Japan; 2 Department of Radiology, Kitasato University Kitasato Institute Hospital, Minato-ku, Tokyo, Japan; 3 Division of Gastroenterology and Hepatology, Department of Internal Medicine, Keio University School of Medicine, Shinjuku-ku, Tokyo, Japan; 4 Department of Gastroenterology and Hepatology, Kitasato University Kitasato Institute Hospital, Minato-ku, Tokyo, Japan; University Hospital Llandough, UNITED KINGDOM

## Abstract

**Background/aims:**

The small bowel is affected in more than half of patients with Crohn’s disease (CD) at the time of diagnosis, and small bowel involvement has a negative impact on the long-term outcome. Many patients reportedly have active lesions in the small intestine even in patients in clinical remission. This study was performed to compare findings of magnetic resonance enterography (MRE) and ileocolonoscopy.

**Methods:**

A single-center retrospective study was conducted in 50 patients (60 imaging series) with CD, for whom MRE was additionally performed during the bowel preparation for subsequent ileocolonoscopy. Endoscopic remission was defined as a Simple Endoscopic Score for CD (SES-CD) of <5. MRE remission was defined as a Magnetic Resonance Index of Activity (MaRIA) score of <50. The time to treatment escalation was assessed by the log-rank test.

**Results:**

Importantly, 7 of 29 patients (24.1%) with endoscopic remission had a MaRIA score of ≥50. Both SES-CD and MaRIA correlated with the need for treatment escalation (*P* = 0.025, *P* = 0.009, respectively). MRE predicted the need for treatment escalation even in patients with endoscopic remission. Although no correlation was present between SES-CD and MaRIA score in patients with structuring/penetrating disease, or insufficient ileal insertion (<10cm), a high MaRIA score still correlated with the need for treatment escalation in stricturing or penetrating disease (*P* = 0.0306).

**Conclusions:**

The MaRIA score predicts the need for treatment escalation even in patients with endoscopic remission, indicating that addition of MRE to conventional ileocolonoscopy alone can be a useful, noninvasive tool for monitoring CD especially in stricturing or penetrating disease.

## Introduction

Crohn’s disease (CD) is a chronic inflammatory disease of the gastrointestinal tract that may flare and remit over time, and the number of affected patients is increasing. Persistent inflammation results in disease progression due to structural bowel damage, which often requires surgical bowel resection. Therefore, it is important to closely monitor the disease activity and achieve sustained remission and mucosal healing to prevent the progression of bowel damage.

The small bowel is reportedly affected in 53% of patients with CD at the time of diagnosis (ileal and ileocolonic involvement in 27% and 26% of patients, respectively) according to the Montreal classification[1]. Small bowel involvement increases to 61% after 5 years[1]. The assessment of small intestinal lesions is important; one study showed that 43% to 60% of patients with established CD had suspected small bowel involvement[2]. Small bowel involvement negatively affects the long-term outcome of CD but is less associated with C-reactive protein and fecal calprotectin[3–6]. Ileal involvement in patients with CD is a significant risk factor[7, 8].

Conventional ileocolonoscopy is a valuable tool in the assessment of CD; it is widely accessible, however, it may not be sufficient to evaluate the entire small bowel involvement[9]. Balloon-assisted enteroscopy enables observation beyond the reach of conventional ileocolonoscopy, but it requires specific devices and highly trained endoscopists[2]. Capsule endoscopy is another endoscopic tool but not an easy first-line examination because of the risk of capsule retention in patients with stricturing disease[10]. These endoscopic methods enable to accurately detect the luminal inflammation with high sensitivity[11], whereas their common weakness can be related to the transmural nature of inflammation caused by CD[12]. MRE is a noninvasive cross-sectional examination technique that may overcome this weakness of endoscopy, but it is less sensitive than endoscopy in detecting stenosis and superficial inflammation[6, 13–15].

Taken together, we hypothesized that adding MRE to conventional ileocolonoscopy during the bowel preparation might be beneficial to compensate their weakness. In the present study, we retrospectively compared findings of ileocolonoscopy and MRE to evaluate the possible usefulness of this procedure.

## Methods

### Patients

We performed a retrospective chart review of 365 series of ileocolonoscopy and 140 series of MRE conducted in patients with CD from January 2013 to November 2017 at Kitasato University Kitasato Institute Hospital. Fifty patients (60 imaging series) in whom both examinations were performed on the same day were included in this study. The patients underwent evaluation of clinical severity (CD activity index [CDAI] and Harvey–Bradshaw Index [HBI])[16, 17], measurement of laboratory parameters including C-reactive protein (CRP), and performance of ileocolonoscopy combined with MRE. Treatment escalation was defined by the addition of corticosteroids, anti-tumor necrosis factor agents, immunomodulators, and surgery. The need for treatment escalation was determined based on the attending physician’s evaluation of aggravated symptoms in the patients. Patients with an ileoanal pouch or ileorectal anastomosis were excluded. Ileocolonoscopy and MRE were performed to assess the severity, location, and extent of disease or to carry out clinical follow-up of CD.

### Ethical consideration

The Research Ethics Committee of Kitasato University Kitasato Institute Hospital approved the study protocol and all documents (approval number: 17042).

### Endoscopic procedure

All patients ingested 1000 mL of polyethylene glycol (PEG) before MRE. After MRE, the patients were required to orally ingest 0 to 1000 mL of additional PEG for a total of 1000 to 2000 ml as a standard bowel preparation regimen for ileocolonoscopy. A long slim colonoscope (PCF-PQ260L; Olympus Medical Systems, Tokyo, Japan) was routinely utilized to enable deeper insertion through possible strictures in daily clinical practice for patients with CD. All segments were retrospectively and separately scored by the Simple Endoscopic Score for CD (SES-CD), and the scores of each segment were calculated to include the sum of the scores in five segments[18]. A segment was scored as 0 if it could not be reached by ileocolonoscopy. Endoscopic remission was defined as an SES-CD of <5[19]. SES-CD was retrospectively scored blinded to the results of MRE.

### MRE procedure and evaluation of CD

All patients were instructed to orally ingest 1000 mL of PEG within 45 to 60 minutes before MRE. MRE was performed using a 1.5-T magnetic resonance imaging unit (Signa HDx; GE Healthcare, Tokyo, Japan). Patients were placed in the supine position on the magnetic resonance imaging table using a previously described protocol[20] (S1 Table). The presence of stricture, fistulae, and abscesses was also assessed on MRE. Relative contrast enhancement (RCE) was calculated as previously reported[13].

The images were retrospectively evaluated by a radiologist (N.K) with more than 10 years of experience blinded to the clinical information and the results of the endoscopic examination. The severity and extent of inflammatory lesions were evaluated using the Magnetic Resonance Index of Activity (MaRIA) score[18, 21]. The overall MaRIA score was calculated as the sum of MaRIA in six segments (distal ileum, ascending, transverse, descending, sigmoid colon, and rectum). MRE remission was defined as a MaRIA score of <50[22]. Restricted maximum likelihood estimation is performed using all of the data in the MaRIA score, even if missing values are present. Multiple imputation was used to impute overall MaRIA for those missing data[23, 24].

### Statistical methods

The mean and standard deviation or the median and range were calculated for parametric and nonparametric data, respectively. Between-group differences were evaluated for time to treatment escalation. Continuous data are summarized as median and interquartile range (IQR), while categorical data are summarized as count and percentage. Nonparametric Spearman rank correlation (r_s_) was used to assess continuous and ordinal bivariate relationships between MRE findings (MaRIA score) and endoscopic findings (SES-CD). A Kaplan–Meier evaluation was carried out to compare treatment escalation between the two groups of patients, with identified differences evaluated using the log-rank statistics. A Cox proportional hazards model was then applied to assess the time to treatment escalation. A *P*-value of <0.05 was considered significant, and correction by Bonferroni’s method was applied when needed. All statistical analyses were carried out using the JMP software program, version 14.1 (SAS Institute, Cary, NC, USA).

## Results

### Characteristics of the study population

The patients’ characteristics are presented in Table 1 (34 men, 16 women). Their median age was 34.5 years (IQR, 26.0–42.0 years). The median follow-up period was 449 days (IQR, 183–853.75 days). No serious adverse events related to either MRE or ileocolonoscopy were observed.

**Table 1:** Patients’ characteristics (*n* = 50).

Sex	
Male	34 (68.0)
Female	16 (32.0)
Age, years	34.5 (26.0–42.0)
Montreal classification	
Age at diagnosis	
<17 years (A1)	11 (22.0)
≥17 to ≤40 years (A2)	33 (66.0)
>40 years (A3)	6 (12.0)
Behavior	
Nonstricturing, nonpenetrating (B1)	21 (42.0)
Stricturing (B2)	15 (30.0)
Penetrating (B3)	14 (28.0)
Disease location	
Ileum only (L1)	16 (32.0)
Colon only (L2)	3 (6.0)
Ileum and colon (L3)	31 (62.0)
Proximal GI tract (L4)	6 (12.0)
Perianal GI tract (p)	12 (24.0)
Body mass index, kg/m2	20.8 (18.7–22.8)
Surgical history	
Appendectomy	10 (20.0)
Crohn’s disease-related surgery	20 (40.0)
Duration of disease, mo	108 (10.5–226)
CDAI	113.7 ± 73.2
Harvey–Bradshaw Index	2.0 ± 2.3
Overall MaRIA	37.9 (29.1–51.5)
SES-CD	5 (0–11)
Medication profile	
5-Aminosalicylic acid	37 (74.0)
Glucocorticoid	6 (12.0)
Azathioprine	10 (20.0)
6-Mercaptoprine	14 (28.0)
Infliximab	12 (24.0)
Adalimumab	3 (6.0)
Elemental diet	16 (32.0)
Antibiotics	3 (6.0)
None	3 (6.0)
Laboratory tests	13.1 ± 1.9
Hemoglobin, g/dL	0.15 (0.04–1.01)
CRP, mg/dL	
Smoking habits	
Active smoking	5 (10.0)
Past smoking	11 (22.0)
Never smoking	34 (68.0)
Family history of Crohn’s disease	4 (8.0)

Data are presented as n (%), median (interquartile range), or mean ± standard deviation.

GI, gastrointestinal; CDAI, Crohn’s Disease Activity Index; the Magnetic Resonance Index of Activity, MaRIA; the Simple Endoscopic Score for CD, SES-CD; CRP, C-reactive protein.

The CDAI consists eight factors, with each factor totaled after adjustment using a weighing factor ranging from 1 to 30. The CDAI ranges from approximately 0 to 600, with higher scores indicating more severe disease activity.

The glucocorticoids included budesonide.

### Relationship between SES-CD and MaRIA score

**Fig 1:**
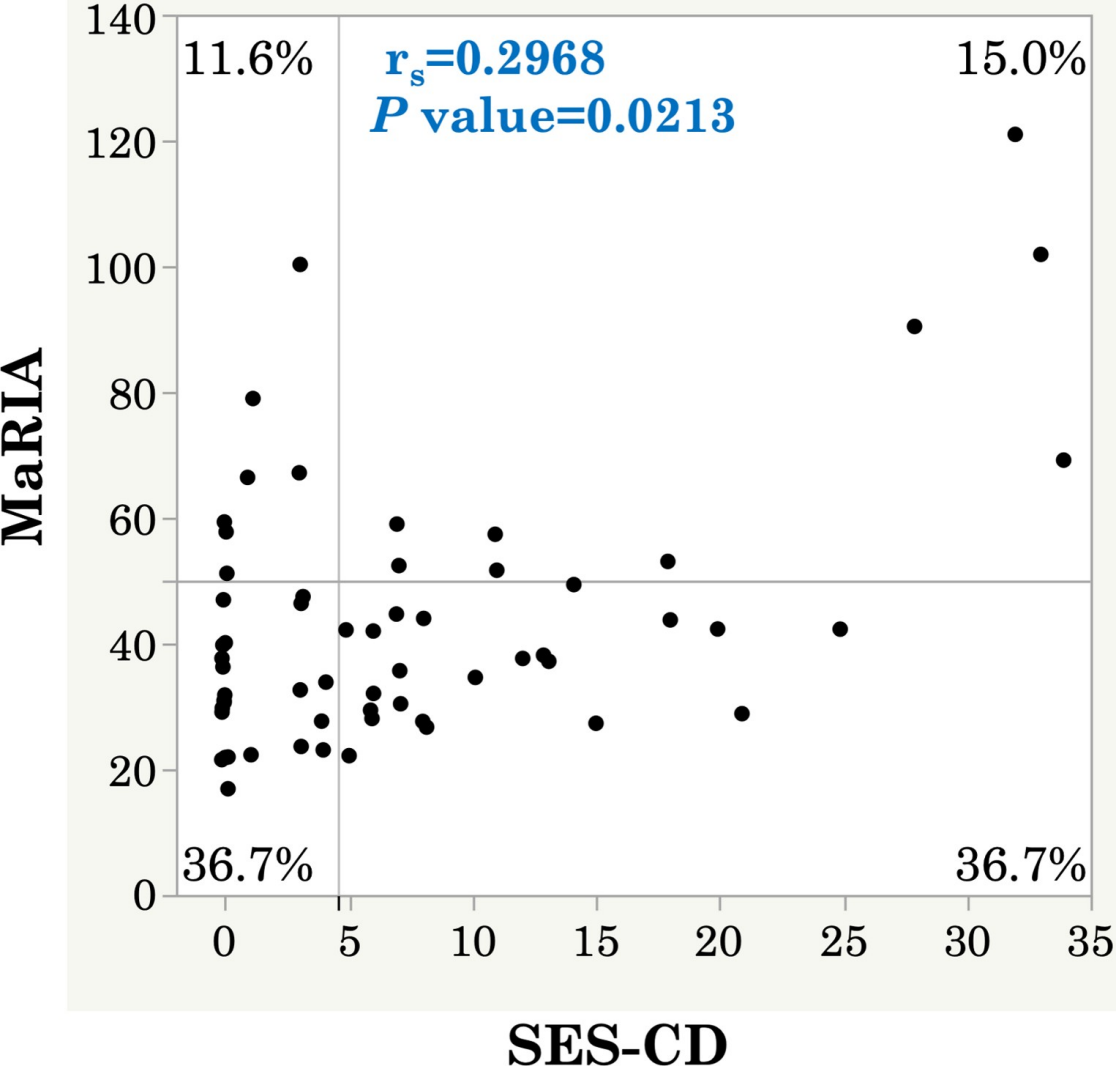
Correlation between SES-CD and MaRIA score (per patient).

Next, we evaluated the relationship between the SES-CD and MaRIA score. The Spearman rank correlation coefficient between the SES-CD and MaRIA score showed a low significant correlation (*r*_*s*_ = 0.2968, *P* = 0.0213) (Fig 1). The correlations between the segmental SES-CD and segmental MaRIA score in six segments were shown in S1 Fig. Importantly, 7 of 29 patients (24.1%) with a negative SES-CD had an overall MaRIA score of ≥50.

### SES-CD and MaRIA score are associated with a time to treatment escalation

**Fig 2:**
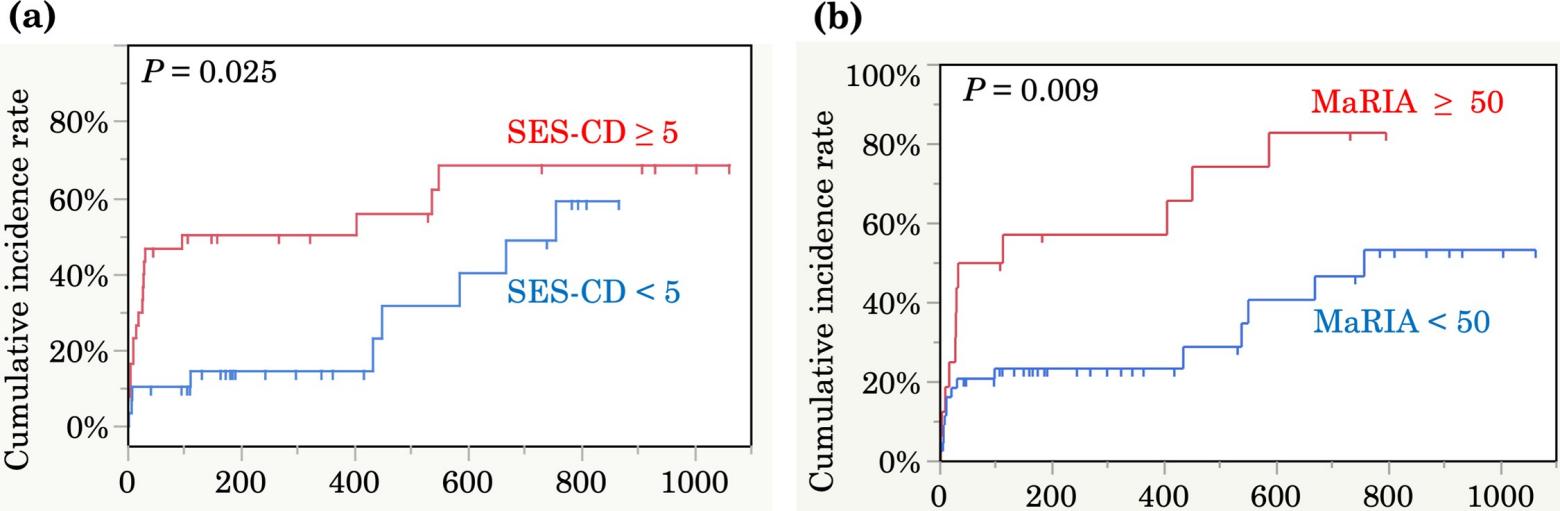
Kaplan–Meier estimates of the cumulative incidence of treatment escalation according to endoscopic findings and MRE findings. (a) Patients with endoscopic remission (blue line, *n* = 29) showed a significantly longer time to treatment escalation than patients with endoscopically active disease (red line, *n* = 31) (*P* = 0.025). (b) Patients with a low MaRIA score (blue line, *n* = 44) showed a significantly longer time to treatment escalation than patients with a high MaRIA score (red line, *n* = 16) (*P* = 0.009).

Kaplan–Meier curves revealed a longer time to treatment escalation in patients with endoscopic remission (SES-CD of <5) than in patients without endoscopic remission (SES-CD of ≥5) (hazard ratio, 2.43; 95% confidence interval, 1.09–5.42; *P* = 0.0302) (Fig 2A). Furthermore, active disease in MRE (MaRIA score of ≥50) was associated with a higher incidence of treatment escalation than was disease remission (MaRIA score of <50) (hazard ratio, 2.65; 95% confidence interval, 1.23–5.70; *P* = 0.0121) (Fig 2B).

### Differential roles of ileocolonoscopy and MRE

**Fig 3:**
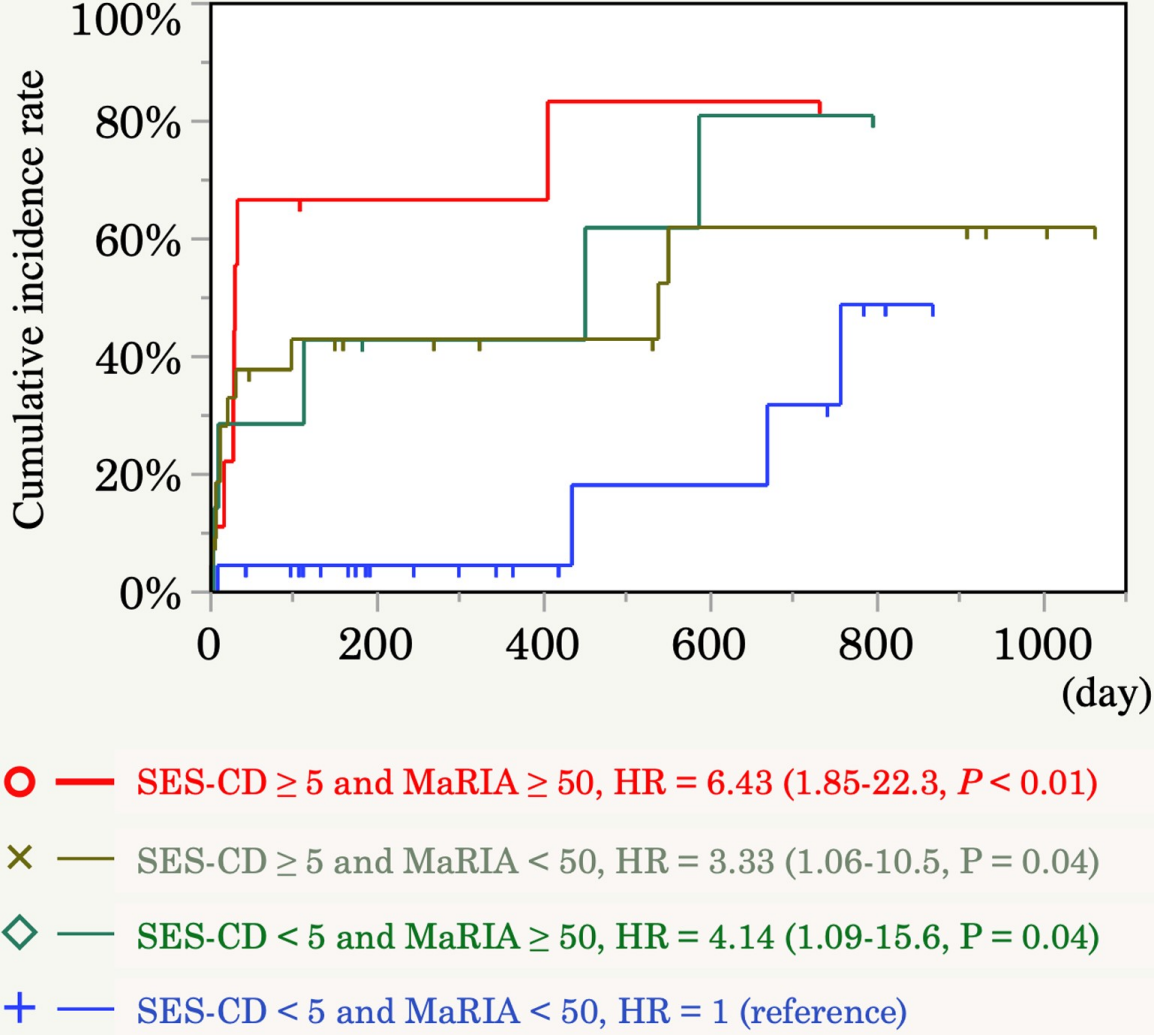
Kaplan–Meier estimates of the cumulative incidence of treatment escalation according to MRE findings and endoscopic findings. Probability of treatment escalation according to active disease in both ileocolonoscopy and MRE (red line, *n* = 9), active disease only in MRE (green line, *n* = 7), active disease only in ileocolonoscopy (gray line, *n* = 22), or remission in both ileocolonoscopy and MRE (blue line, *n* = 22). A low MaRIA score was associated with a longer incidence-free duration even in patients with endoscopic remission. Hazard ratio (HR) with 95% confidence interval (CI) and *P* value are shown. Blue line (both remission finding in SES-CD and MaRIA) represents control group).

**Fig 4:**
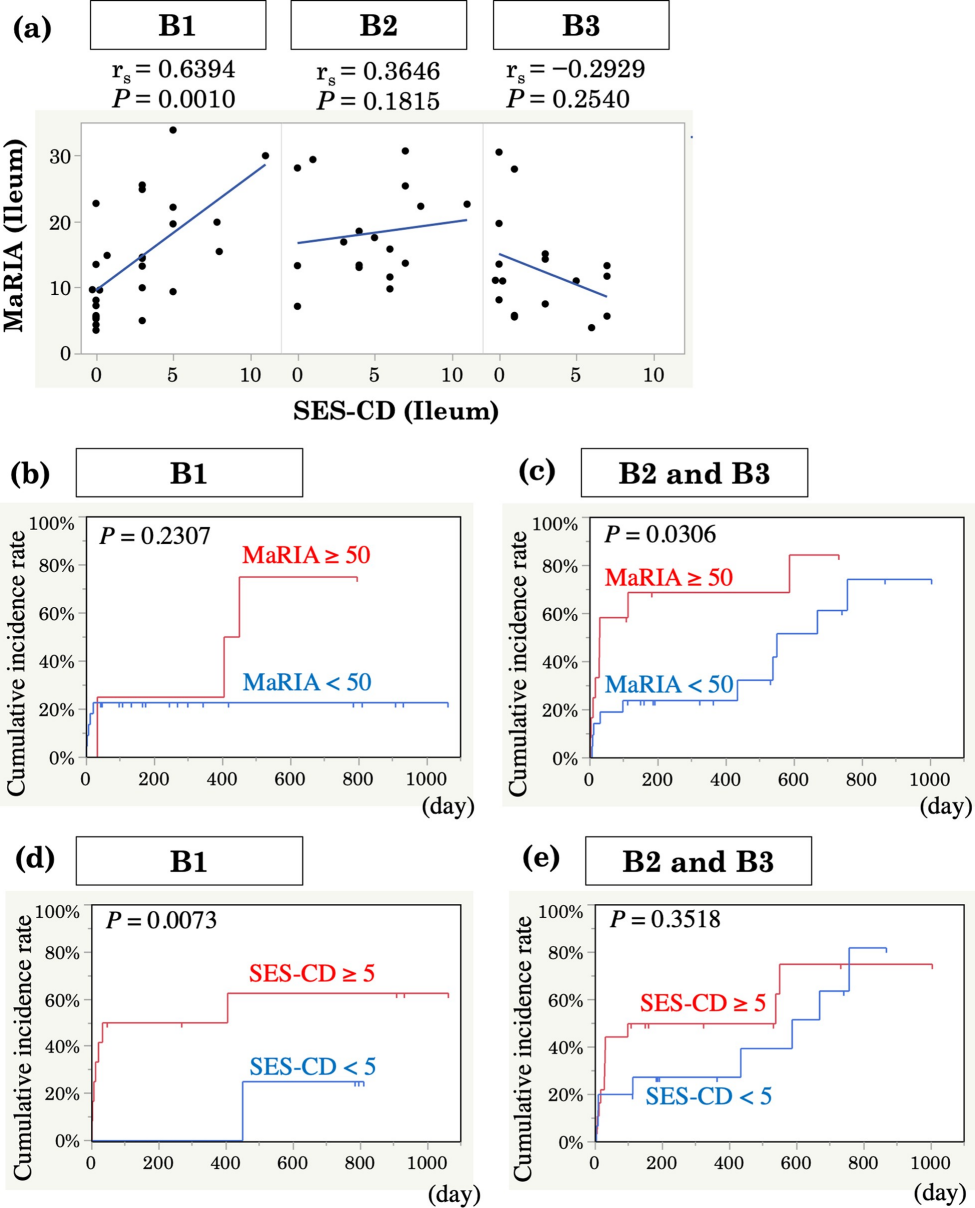
Usefulness of MRE in predicting early need for treatment escalation in patients with stricturing or penetrating disease. (a) Correlation between the SES-CD and MaRIA score in the distal ileum according to the Montreal classification. (b) Kaplan–Meier estimates of the cumulative incidence of treatment escalation according to MRE findings in patients with B1 (without strictures or penetrating disease, *n* = 26) or (c) B2/3 (with strictures or penetrating disease, *n* = 34) disease. (d) Kaplan–Meier estimates of the cumulative incidence of treatment escalation according to endoscopic findings in patients with B1 or (e) B2/3 disease.

**Fig 5:**
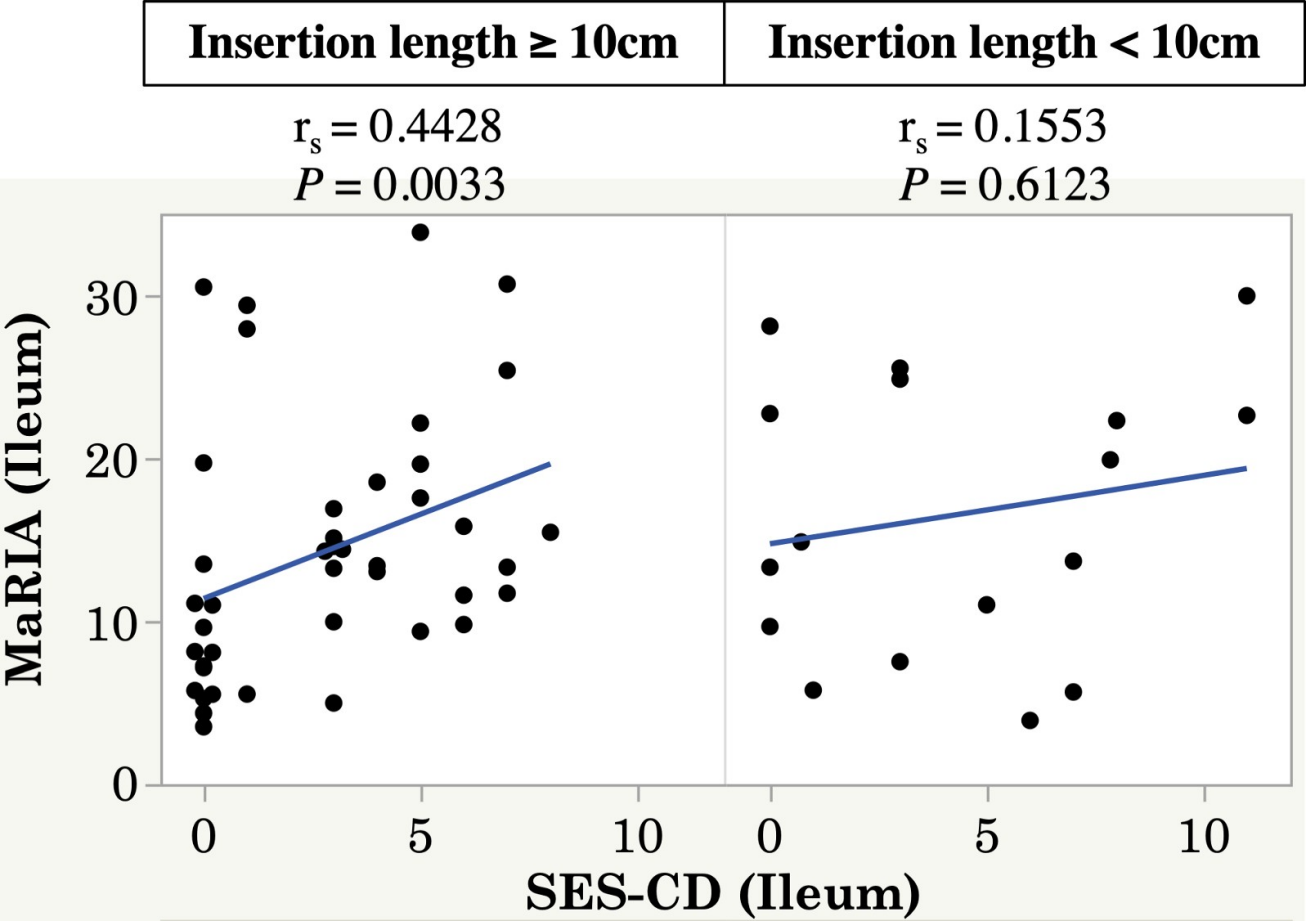
SES-CD correlated with MaRIA score when ileocolonoscopy was inserted >10cm. Correlation between the SES-CD and MaRIA score in the distal ileum according to insertion length. (with observing 10 cm or more of distal ileum, *n* = 17) or (with observing less than 10 cm of distal ileum, *n* = 43).

Since there were some patients still needed treatment escalation despite SES-CD<5 or MaRIA<50 (Fig 2A and 2B). we next assessed combined efficacy in predicting the need for treatment escalation. Patients with positive findings in both endoscopy and MRE showed the highest need for treatment escalation, indicating that both MRE and endoscopic findings are important (hazard ratio, 6.43; 95% confidence interval, 1.85–22.3; *P* <0.01) (Fig 3). The influence of active lesions in endoscopy in patients with MRE remission is shown in Fig 3 (hazard ratio, 3.33; 95% confidence interval, 1.06–10.5; *P* = 0.04). Active disease on MRE (defined as a MaRIA score of ≥50) was associated with a shorter time to treatment escalation, even in patients with endoscopic remission (hazard ratio, 4.14; 95% confidence interval, 1.09–15.6; P = 0.04) (Fig 3). Next, we investigated the correlation between the SES-CD and MaRIA score in each disease category according to the Montreal classification to explain the discrepancy between the SES-CD and MaRIA score. The Spearman rank correlation coefficient between the SES-CD and MaRIA score was strong (r_s_ = 0.6394) (*P* = 0.001) in patients with inflammatory disease (B1), whereas there was no correlation in patients with stricturing or penetrating disease (B2, B3) (Fig 4A). A high MaRIA score predicted the early need for treatment escalation in patients with lesions (B2, B3) (Fig 4B and 4C), while a high SES-CD in patients with B1 disease was associated with the early need for treatment escalation but not in patients with B2/B3 disease (Fig 4D and 4E). In addition, we found that SES-CD significantly correlated with MaRIA when ileal insertion was sufficient (**≥** 10cm) (Fig 5), suggesting the importance of lesions beyond the reach of colonoscopy.

## Discussion

In this study, we retrospectively compared ileocolonoscopy and MRE findings and assessed the usefulness of adding MRE during bowel preparation for ileocolonoscopy.

Clinical and serological remission cannot exclude the presence of active lesions in MRE, ileocolonoscopy balloon-assisted enteroscopy, or video capsule endoscopy [6, 25]. Thus, clinical symptoms and the CRP level are not sensitive enough to detect active lesions in patients with CD[5, 6, 26, 27].

MRE is one of the most widely used imaging techniques for monitoring small intestinal CD lesions, predicting the outcome of CD, and helping physicians to make clinical decisions[6, 28, 29]. According to a previous study, it has poorer sensitivity than endoscopy for detecting active lesions[13]. However, a recent study revealed that not only ulcers but also milder lesions such as erosions and redness can be detected in MRE by adding relative contrast enhancement and diffusion-weighted imaging[30]. Evaluation of MaRIA add the presence of disease activity from endoscopic activity[12]. These previous findings support our data showing that MRE is considered a useful additional diagnostic technique, particularly in patients with transmural inflammation.

The accuracy of MRE for assessment of CD location, activity and complications has been confirmed using CDEIS, SES-CD, clinical index, histopathological findings, and panel diagnosis as reference standards[6, 22, 31, 32]. However, in our study, the correlation between SES-CD and MaRIA in the entire cohort was not strong. There are a few possible reasons that could explain this discrepancy. First, we have more patients with stricturing/penetrating disease compared with previous publications[21, 22, 33]. As shown in Fig 4, MaRIA still correlated with SES-CD in B1 disease but not in B2/3. Furthermore, SES-CD predicted need for treatment escalation in MRE-negative B1 disease, while MaRIA was superior to ileocolonoscopy in predicting need for treatment escalation in B2/3 disease. This is clinically relevant considering the cross-sectional feature of MRE and its capability of assessing transmural inflammation. Second possible explanation is sufficient observation of distal ileum by MRE when the endoscope cannot be inserted deep enough. Our analysis showed the discrepancy between segmental SES-CD and MaRIA score in the distal ileum when the insertion was less than 10cm from the ileocecal valve. Third, we did not give any transanal preparation as previously proposed using warm water enema. This might have worsened the accuracy of MRE in the distal colorectum and caused this discrepancy[34]. Poorly distended segments may mimic bowel wall thickening or mucosal hyperenhancement, thereby falsely assessing the presence of inflammatory changes[34]. In some cases, elimination of solid stools was not enough to get precise details of the mucosa. However, this should be overcome by ileocolonoscopy combined with MRE in our procedure.

A discrepancy between ileocolonoscopy and MRE findings may in fact justify combination of ileocolonoscopy and MRE. Our study shows that positive findings in MRE predict treatment escalation in patients with negative ileocolonoscopy findings. Therefore, in clinical practice, the combination of ileocolonoscopy and MRE might be useful especially in patients at high risk of intestinal complications. This finding is inconsistent with a previous study showing that MRE is less sensitive in detecting strictures[33]. These differences in patients with stricturing or penetrating disease can be explained by the scoring system for the SES-CD, in which the presence of a stricture increases the score. Using the SES-CD active score with exclusion of the scoring item “strictures” from the original SES-CD might be useful to eliminate this discrepancy[14, 18]. Because the proportion of patients with intestinal complications (B2, B3) increases over time[35], the combination of ileocolonoscopy and MRE might be highly significant for patients with CD exhibiting a long disease duration.

Our bowel preparation regimen for ileocolonoscopy requires 2 L of PEG; thus, MRE can be performed during preparation without additional PEG intake. Performing the two methods in combination has various advantages. First, MRE may be able to detect ileal lesions that cannot be observed by ileocolonoscopy as well as transmural lesions. Second, double-checking of the distal ileum by both ileocolonoscopy and MRE may help to screen the distal ileum, which CD most frequently affects, with a higher sensitivity. Third, ileocolonoscopy allows for detailed mucosal observation and histopathological evaluation. Fourth, the ability to perform the procedure in 1 day may improve the patient’s acceptability by reducing the burden of bowel preparation. Undergoing bowel preparation twice (before both ileocolonoscopy and MRE) was considered burdensome and performing both endoscopy and MRE in a short period of time contributed to lower acceptance of repeated examinations in previous studies[22, 36]. Our 1-day procedure might relieve the burden of undergoing ileocolonoscopy and MRE on different days.

The current study has some limitations. The study is susceptible to limitations inherent to the retrospective design. Whether abnormal findings lead to additional therapy or additional therapy was added in the future in patients with abnormal findings remains uncertain. In addition, patient selection might include the selection bias; we cannot fully exclude the possibility that enrolled patients received this combination of ileocolonoscopy and MRE when they were considered treatment escalation for other reasons. The other limitation is the lack of fecal biomarkers in our study. Increasing evidence suggests that fecal calprotectin is useful in monitoring CD activity and predicting relapse[37]. We used ileocolonoscopy and MRE but used neither balloon-assisted enteroscopy nor capsule endoscopy. Both examination techniques have risk of false-negative results in the jejunum, and ileocolonoscopy has a risk of false-negative results in the ileum or more proximal small bowel because they are out of reach.

## Conclusions

The present study suggests the possible benefit of combining MRE with ileocolonoscopy in patients with complicated CD. A prospective study with a larger sample size and more clinically relevant endpoints, such as the rate of hospitalization and operation rate, is warranted.

## Supporting information

S1 Fig Correlation between SES-CD and MaRIA score (per segment). (TIFF)

S1 Table Parameter of MR imaging. (DOCX)
