# Supplementary figures and images for: Combination of colonoscopy and magnetic resonance enterography is more useful for clinical decision making than colonoscopy alone in patients with complicated Crohn's disease
Source: PLoS One. 2019 Feb 20;14(2):e0212404. doi: 10.1371/journal.pone.0212404 (PMC6382266; doi:10.1371/journal.pone.0212404)

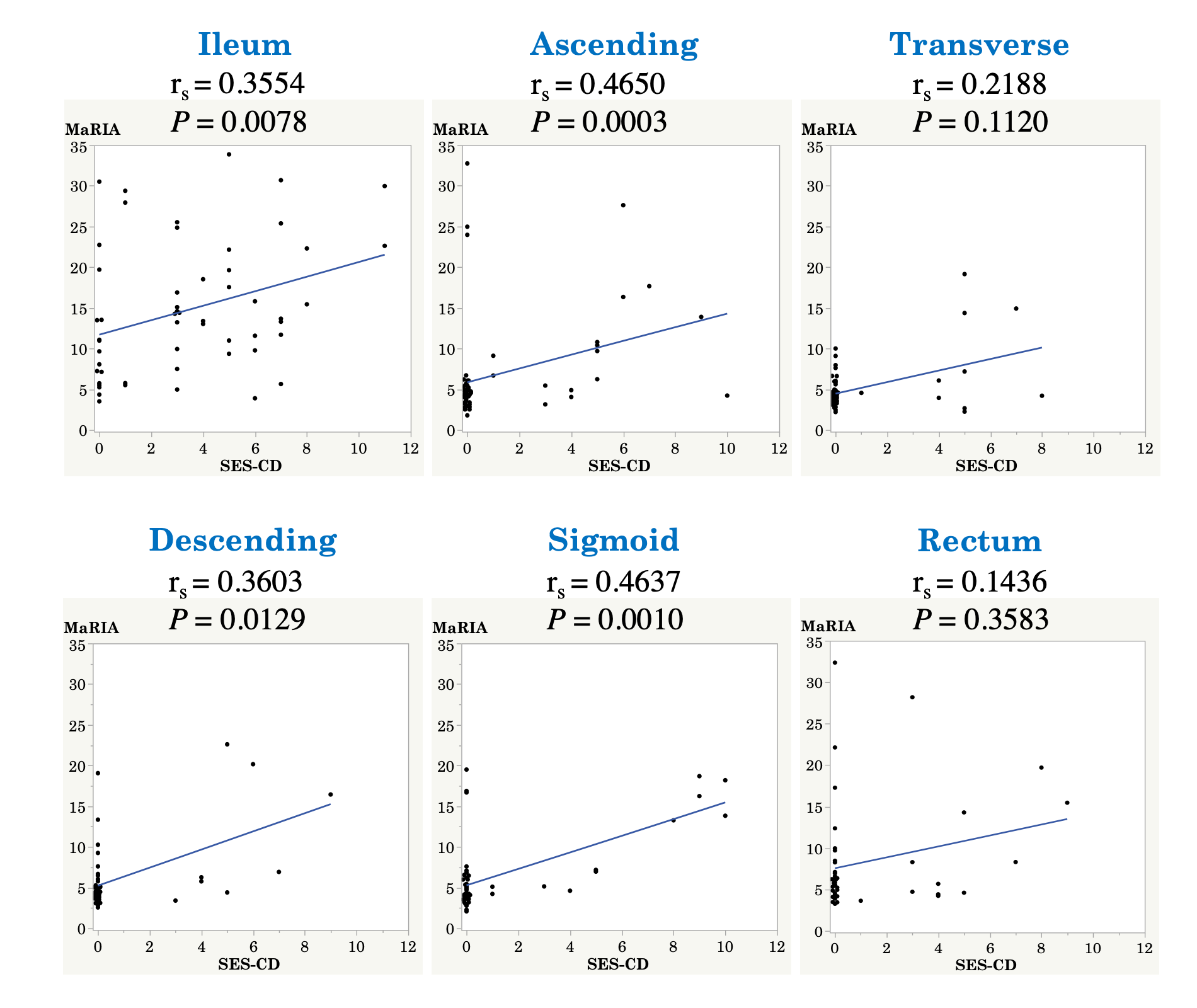

Supplement: S1 Fig — (TIFF) [file pone.0212404.s001.tiff]
