# Supplementary material for: Combination of colonoscopy and magnetic resonance enterography is more useful for clinical decision making than colonoscopy alone in patients with complicated Crohn's disease
Source: PLoS One. 2019 Feb 20;14(2):e0212404. doi: 10.1371/journal.pone.0212404 (PMC6382266; doi:10.1371/journal.pone.0212404)
Supplement: S1 Table — (DOCX) [file pone.0212404.s002.docx]

**S1 Table. Parameter of MR imaging**

| Parameter | MR sequence | | | |
| --- | --- | --- | --- | --- |
|  | SSFSE | | LAVA | |
| section orientation | Axial | Coronal | Axial | Coronal |
| TR/TE (ms) | ∞/90 | ∞/100 | 3.6/1.7 | 2.8/1.4 |
| flip angle | 90 | 90 | 15 | 15 |
| fat saturation | Yes | No | Yes | Yes |
| Acceleration Factor | 2 | 2 | 2 | 2 |
|  | (ASSET) | | | |
| matrix size | 288x192 | 384x160 | 256x224 | 256x256 |
| field of view | 36 | 48 | 40 | 44 |
| section thickness | 7 | 5 | 5 | 4 |
| section gap | 3 | 1 | 0 | 0 |

SSFSE (Single Shot Fast Spin Echo)

LAVA (Liver Acquisition with Volume Acceleration)

ASSET (Array Spatial Sensitivity Encoding Techniques): image domain parallel imaging

ARC (Autocalibrating Reconstruction for Cartesian parallel imaging): *K*-space parallel imaging
